# Elevated cytokine levels in the central nervous system of cluster headache patients in bout and in remission

**DOI:** 10.1186/s10194-024-01829-9

**Published:** 2024-07-23

**Authors:** Caroline Ran, Felicia Jennysdotter Olofsgård, Katrin Wellfelt, Anna Steinberg, Andrea Carmine Belin

**Affiliations:** 1 Centre for Cluster Headache, Department of Neuroscience, Karolinska Institutet, Stockholm, Sweden; 2 Department of Clinical Neuroscience, Karolinska Institutet, Stockholm, Sweden; 3 Department of Neurology, Karolinska University Hospital, Stockholm, Sweden

**Keywords:** Inflammation, Neuroinflammation, Headache, Chemokines

## Abstract

**Background:**

Cluster headache is characterized by activation of the trigeminovascular pathway with subsequent pain signalling in the meningeal vessels, and inflammation has been suggested to play a role in the pathophysiology. To further investigate inflammation in cluster headache, inflammatory markers were analysed in patients with cluster headache and controls.

**Methods:**

We performed a case–control study, collecting cerebrospinal fluid and serum samples from healthy controls, cluster headache patients in remission, active bout, and during an attack to cover the dynamic range of the cluster headache phenotype. Inflammatory markers were quantified using Target 48 OLINK cytokine panels.

**Results:**

Altered levels of several cytokines were found in patients with cluster headache compared to controls. CCL8, CCL13, CCL11, CXCL10, CXCL11, HGF, MMP1, TNFSF10 and TNFSF12 levels in cerebrospinal fluid were comparable in active bout and remission, though significantly higher than in controls. In serum samples, CCL11 and CXCL11 displayed decreased levels in patients. Only one cytokine, IL-13 was differentially expressed in serum during attacks.

**Conclusion and interpretation:**

Our data shows signs of possible neuroinflammation occurring in biological samples from cluster headache patients. Increased cerebrospinal fluid cytokine levels are detectable in active bout and during remission, indicating neuroinflammation could be considered a marker for cluster headache and is unrelated to the different phases of the disorder.

**Supplementary Information:**

The online version contains supplementary material available at 10.1186/s10194-024-01829-9.

## Introduction

Cluster headache (CH) is a trigeminal autonomic cephalalgia characterized by recurring unilateral headache attacks of extreme severity. Patients with CH typically experience active bouts where they can have up to eight attacks per day, followed by symptom free remission periods [1]. The trigeminovascular pathway and the trigeminal–autonomic reflex are activated during CH attacks, resulting in severe pain in the periorbital region as well as the autonomic symptoms manifesting during CH attacks. Nevertheless, the underlying mechanisms are unclear and CH pathophysiology remains to be fully elucidated [2].

Inflammatory reactions have been suggested in the past to cause CH, in particular those of the cavernous sinus [3], and the corticosteroid prednisolone, a powerful anti-inflammatory drug, is commonly used as an interim treatment for CH [4]. Meanwhile, there is no evidence of systemic inflammation in CH patients [5]. Targeted analysis of inflammatory markers in CH has yielded inconclusive results, often because of small sample sizes and usage of different methodologies. In a large microarray panel screening for differentially expressed genes in peripheral blood, inflammation related genes were found to be upregulated in CH patients as compared to controls (major histocompatibility complex, class II (*HLA*) *-DQA1* and *HLA-DQB1*), or specifically during active bouts (S100 calcium binding protein (*S100*) A8 and *S100A12*) [6]. In another study, a trend for interleukin (*IL*) -1β and nuclear factor-κB (*NF-κB*) upregulation was found in blood cells from patients with CH in active bout [7]. Interestingly, the authors found a concurrent reduction in inflammasome component NLR family pyrin domain containing 3 (*NLRP3*) expression levels, required for IL-1β release [7]. In another global gene expression analysis in peripheral blood cells, inflammatory processes were highlighted both by pathway analysis (*IL-4* and sialic acid binding Ig like lectin 7 (*CD328*)) and by expression network analysis as potentially involved in CH [8]. Increased gene expression of *IL-2* has further been reported in CH patients, specifically in active bout in between CH attacks, while mRNA levels were normalized to control levels during attacks [9]. Reports on differentially expressed cytokines in CH are scarce, IL-2, and IL-1β have been suggested to be increased in CH patients, but a recent meta-analysis could not confirm elevated IL-1β [10–12]. The same meta-analysis summarized findings on the more common primary headaches migraine and tension type headache (TTH) and confirmed higher levels of IL-6, IL-8 and tumour necrosis factor alpha (TNF-α) in migraine patients and of TNF-α and transforming growth factor beta (TGF-β) in TTH compared to controls [11].

Even less is known regarding inflammation of the nervous system in CH. Due to recent developments in CH genetics there have been speculations of microglia involvement in the pathogenesis of CH. Microglia have been linked to CH through genome wide association studies (GWASs) highlighting the gene MER proto-oncogene, tyrosine kinase (*MERTK*), a gene highly expressed in microglia and macrophages involved in phagocytosis of apoptotic cells [13]. In this study we aimed to perform inflammatory profiling of CH patients in remission period and in active bout in order to get a better understanding of the importance of inflammation in CH and if inflammatory reactions may constitute a hallmark of the phenotypic switch occurring in CH patients when they go into active bout.

## Methods

### Material

Study participants between the ages of 18 and 65 were recruited at the neurology clinic at Karolinska University Hospital, 34 patients diagnosed with episodic CH and 40 control subjects (see details in Table 1). CH patients were diagnosed by a neurologist (co-author A.S.) according to the international classification of headache disorders (ICHD) 3rd edition [1]. Only patients without other inflammatory disease or acute infections were included in the study. Furthermore, patients were not allowed to use medication with steroids, antibiotics, antiphlogistics, antirheumatics, lithium or verapamil during the current CH period or before sampling. Control subjects were age- and sex-matched patients visiting the neurology clinic at Karolinska University Hospital for other conditions than headache disorders, inflammatory illness or acute infections, and who had not used anti-inflammatory drugs or antibiotics in the last four weeks. Informed consent was obtained from all study participants prior to inclusion. Ethical permit to perform the study was obtained from the Swedish Ethical Review Authority (diary number 217/02) and all experiments were conducted in accordance with the declaration of Helsinki for research involving human subjects.

**Table 1:** Details of the cohort

Disease status	Subjects (n)	Female sex (n)	Average age (years)	Tissue type	Samples (n)	Remission/active bout/active attack (n samples)
Cluster headache	34	35.2% (12)	46.9	CSF	43	18/25/0
				Serum	29	11/15/3
Healthy control	40	31.4% (11)	48.6*	CSF	21	NA
				Serum	27	NA

^*^Based on study participants for whom information was available

*NA* Not applicable

Study participants were asked to give a sample of cerebrospinal fluid (CSF) or serum (blood) or both. Tissue from patients was sampled at several timepoints if possible; in remission (after a minimum of three weeks in remission period), in active bout, and during a CH attack. Venous blood samples were taken during an attack, at the earliest 15 min after the start of a CH attack. In total we collected and analysed 120 samples (Table 1): 21 CSF samples and 27 serum samples from 40 control individuals, 43 CSF samples from 27 CH patients, (18 in remission and 25 in active bout), and 29 serum samples from 21 CH patients, (11 in remission, 15 in active bout and 3 during an attack). Samples were acquired using standard procedures and kept at -80°C until analysis.

### OLINK cytokine panels

Samples (CSF and serum) were analysed, and quality control and calibrator normalization were performed at SciLifeLab Affinity Proteomics Uppsala. We used OLINK® Proximity Extension Assay (PEA) Target 48 cytokine panels, (Affinity Proteomics Uppsala, SciLifeLab, Uppsala University, SE-751 85 Uppsala). The panel contains 45 assays, 89% (*n* = 40) of which passed quality control (assays failing quality control: C-X-C motif chemokine ligand (CXCL) 12, IL-1β, IL-15, IL-17A, and IL-4). 99% (*n* = 119) of the analysed samples passed quality control. Data is reported as normalized protein expression (NPX) in Log2 scale and was analysed separately for CSF and serum samples in R(v4.1.3) using the OlinkAnalyze package.

### Statistical analysis

PCA analysis confirmed that one CSF sample did not pass the quality control and also revealed a cluster of serum samples which may represent outliers (*n* = 7), Supplementary Figs. 1A and 1B. Comparison of group averages between the flagged samples and the remaining controls showed highly heterogenous values, justifying their removal from the analysis. Plotting all samples in a histogram and performing the Shapiro–Wilk test showed that data was not normally distributed, Supplementary Figs. 1C and 1D. Two samples were run in duplicate and removed from the analysis. Assays were removed from analysis if they did not pass quality control and if the number of samples with NPX values below the limit of detection (LOD) was higher than 75%; 11 CSF assays (C–C motif chemokine ligand (CCL) 7, colony stimulating factor 3 (CSF3), interferon gamma (IFN-γ), IL-2, IL-10, IL-13, IL-17F, IL-27, IL-33, TNF-α, thymic stromal lymphopoietin (TSLP)) and 3 serum assays (IL-33, IL-17F, TSLP). Group comparisons were made with one-way ANOVA or Wilcoxon rank-sum test using the Benjamini & Hochberg method for correction for multiple testing. A network analysis of associated cytokines was performed using the STRING database v.12.0 [14].

Post-hoc analysis was performed to understand trends of association in the serum samples. The analysis was run on the entire cohort, on a subset of cytokines comprising a) all cytokines with significant *p*-values before correction for multiple testing, or b) a change in NPX values larger than 10% when comparing CH patients in active bout with controls or when comparing CH patients during attack with CH patients in active bout. The selection resulted in the inclusion of 22 cytokines in the post-hoc analysis.

## Results

The final analysis comprised of 49 serum samples (20 from controls and 29 from CH patients; 11 in remission, 15 in active bout and 3 during an attack) and 61 CSF samples (20 from controls and 41 from CH patients; 17 in remission and 24 in active bout) from 39 control individuals and 34 CH patients in total.

**Fig. 1:**
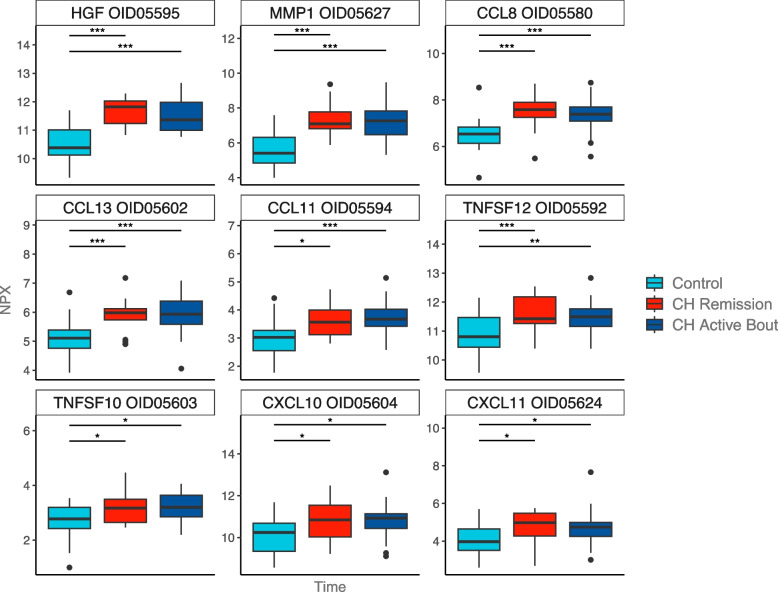
Normalized cytokine expression in CSF from controls and cluster headache patients. Data are represented as NPX, which are normalized protein expression values on a Log2 scale. Analysis comprised 20 controls and 41 CH patients: 17 in remission and 24 in active bout. *: *p*-value < 0.05, **: *p*-value < 0.01, ***: *p*-value < 0.005

**Fig. 2:**
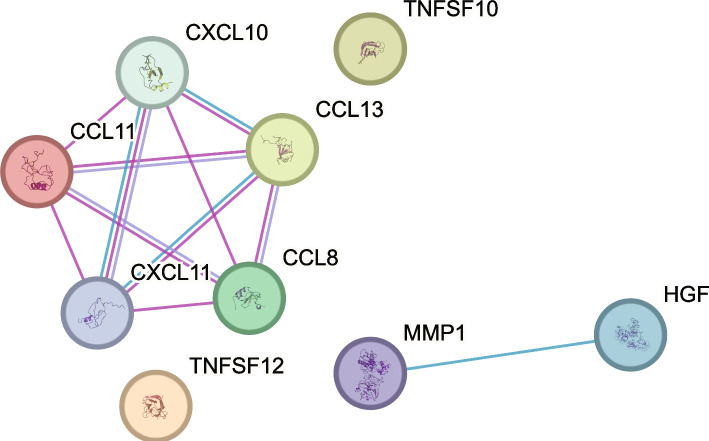
Interactions of cytokines differentially expressed in CSF. STRING network analysis shows known interactions; blue lines represent interactions from curated databases, pink lines represent experimentally determined interactions, purple lines represent protein homology. Number of nodes: 9, number of edges: 11, average node degree: 2.44, avg. local clustering coefficient: 0.778, protein–protein interaction enrichment *p*-value: < 1.0e-16

Analysis of CSF cytokine levels in controls and CH patients showed differences in nine cytokines; CCL8, CCL13, CCL11, CXCL10, CXCL11, hepatocyte growth factor (HGF), matrix metallopeptidase (MMP) 1, TNF superfamily member (TNFSF) 10 and TNFSF12 (Fig. 1, Supplementary Table 1). All nine cytokines were found to be elevated in CSF in CH patients as compared to controls but did not differ between CH patients in remission and in active bout (Fig. 1, Table 2). Network analysis further showed strong interactions between all upregulated chemokines; CCL8, CCL13, CCL11, CXCL10, CXCL11, with a protein–protein interaction enrichment *p*-value < 1.0e-16 (Fig. 2).

**Table 2:** Results from comparison of normalized cytokine expression in controls and cluster headache patients

			Remission		Active		Attack	
Cytokine	Sample	Ref	Estimate	Adj. P	Estimate	Adj. P	Estimate	Adj. P
CCL11	CSF	C	-0.56	**0.016**	-0.73	**<0.001**	NA	NA
	Serum	C	0.98	**0.031**	1.16	**0.003**	-0.14	0.995
CCL13	CSF	C	-0.76	**0.002**	-0.81	**<0.001**	NA	NA
CCL8	CSF	C	-0.97	**<0.001**	-0.90	**<0.001**	NA	NA
CXCL10	CSF	C	-0.78	**0.029**	-0.77	**0.018**	NA	NA
CXCL11	CSF	C	-0.76	**0.028**	-0.70	**0.028**	NA	NA
	Serum	C	1.95	**0.028**	2.42	**0.001**	-0.23	0.997
HGF	CSF	C	-1.11	**<0.001**	-1.00	**<0.001**	NA	NA
MMP1	CSF	C	-1.78	**<0.001**	-1.61	**<0.001**	NA	NA
TNFSF10	CSF	C	-0.55	**0.013**	-0.52	**0.010**	NA	NA
TNFSF12	CSF	C	-0.75	**0.003**	-0.62	**0.007**	NA	NA
CCL11	CSF	Rem	-	**-**	0.17	0.644	NA	NA
	Serum	Rem	-	**-**	-0.18	0.961	1.12	0.248
CCL13	CSF	Rem	**-**	**-**	-0.04	0.976	NA	NA
CCL8	CSF	Rem	**-**	**-**	-0.07	0.947	NA	NA
CXCL10	CSF	Rem	**-**	**-**	0.01	0.999	NA	NA
CXCL11	CSF	Rem	**-**	**-**	-0.06	0.972	NA	NA
	Serum	Rem	**-**	**-**	0.47	0.913	-2.18	0.255
HGF	CSF	Rem	**-**	**-**	0.11	0.817	NA	NA
MMP1	CSF	Rem	**-**	**-**	0.16	0.861	NA	NA
TNFSF10	CSF	Rem	**-**	**-**	0.03	0.985	NA	NA
TNFSF12	CSF	Rem	**-**	**-**	0.12	0.817	NA	NA
CCL11	Serum	Active	**-**	**-**	**-**	**-**	-1.30	0.125
CXCL11	Serum	Active	**-**	**-**	**-**	**-**	-2.65	0.104

*CSF* Cerebrospinal fluid, *Ref* Reference group in analysis, *C* Control, *Rem/Remission* Cluster headache patients in remission, *Active* Cluster headache patients in active bout, *Attack* Cluster headache patients during an attack, *Adj. P* Adjusted *p*-value, *NA* Not Available

**Fig. 3:**
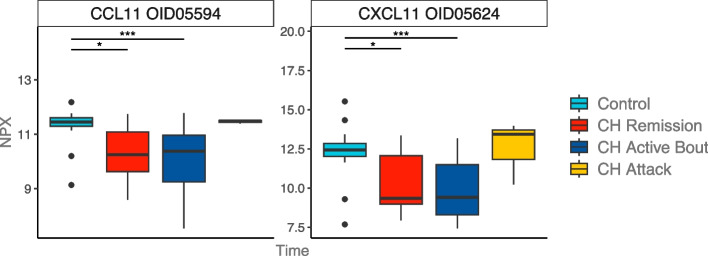
Normalized cytokine expression in serum from controls and cluster headache patients. CH Attack: CH patients in active bout sampled during an attack. Data are represented as NPX, which are normalized protein expression values on a Log2 scale. Analysis comprised 20 controls and 29 CH patients: 11 in remission, 15 in active bout and 3 during an attack. *: *p*-value < 0.05, **: *p*-value < 0.01, ***: *p*-value < 0.005

The analysis of cytokine levels in serum from controls and CH patients in remission, active bout and during a CH attack showed less variation between groups (Supplementary Table 2). Only two cytokines were found to differ between controls and CH patients; CCL11 and CXCL11, both of which were found at lower levels in patients than controls during remission phase and in active bout (Fig. 3). Although lower at baseline, CCL11 and CXCL11 levels in patients were found to raise to control levels during CH attacks. The difference observed between CH patients in remission, or active bout and patients during an attack was not significant, possibly because of a low number of samples (*n* = 3) (Table 2).

### Post hoc analysis of serum cytokines

**Fig. 4:**
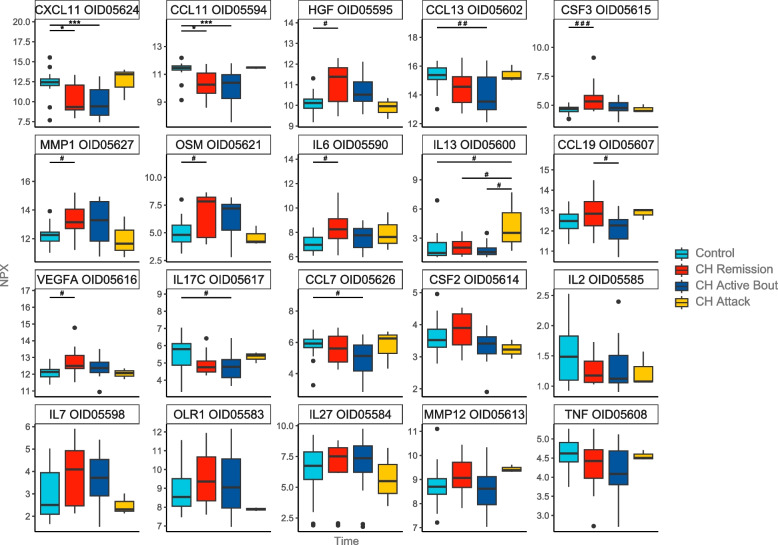
Subthreshold association analysis of cytokines in serum from cluster headache patients and controls. CH Attack: CH patients in active bout sampled during an attack. Data are represented as NPX, which are normalized protein expression values on a Log2 scale. Analysis comprised 20 controls, 11 CH patients in remission, 15 CH patients in active bout and 3 CH patients during an attack. P-values significant after correction for multiple testing in the initial analysis are labelled as follows *: *p*-value < 0.05, **: *p*-value < 0.01, ***: *p*-value. Sub-threshold analysis included 22 cytokines, *p*-values significant after correction for multiple testing in the subthreshold analysis are labelled as follows ^#^: subthreshold association *p*-value < 0.05, ^# #^: subthreshold association *p*-value < 0.01, ^# # #^: subthreshold association *p*-value < 0.005

As the number of patients leaving a sample during a CH attack were few (serum *n* = 3, no CSF samples), the data was revisited comparing assays below significance to understand trends in cytokine levels in serum. Twenty two cytokines were selected for the post-hoc analysis, these were cytokines with significant *p*-values before correction for multiple testing in the primary analysis, or displaying a change in NPX values larger than 10% in active bouts compared to controls or during a CH attack. Data revealed subthreshold associations for several cytokines in serum, variations in four cytokines were of the same direction as the associations detected in the primary analysis (showing decreased levels of CXCL11 and CCL11). Namely CCL13, CCL19, IL-17C and CCL7 which were all displaying trends of decreased levels in CH patients (Fig. 4). Interestingly, these cytokines showed trends of opposite direction in serum as compared to CSF. NPX levels of HGF, CSF3, MMP1, oncostatin M (OSM), IL-6 and vascular endothelial growth factor A (VEGFA) were elevated in CH patients as compared to controls (Fig. 4).

Cytokine levels in samples from CH patients during an attack were consistently comparable with those of controls rather than patients in active bout or remission, with the exception of IL-13, which was detected at higher levels during an attack than in controls and CH patients in remission and in active bout.

## Discussion

We have conducted a large screening of cytokine levels in CH patients and controls using serum and CSF samples in order to get an overview of the inflammatory state in the periphery and in the central nervous system (CNS). Nine cytokines were found to be higher in CSF from patients as compared to controls, particularly cytokines with chemoattractant properties on leukocytes. Interestingly, major differences in cytokine levels were detected between CSF and serum. In the discovery analysis two of the cytokines found at higher levels in CSF were lower in serum.

Previous studies on cytokines in CH have suggested an increase of IL-2 and IL-1β in blood of CH patients although data are conflicting for IL-1β [10, 11]. IL-2 gene expression and protein levels in blood were found to be increased in CH active bout compared to controls, while gene expression levels decreased to control levels during attacks [9, 12]. In our study, the assay for IL-1β did not pass quality control and we could not replicate the findings of an increase for IL-2 in our material. These differences could potentially be due to discrepancies between methodology, quantitative Real-time PCR (qPCR) and enzyme-linked immunosorbent assay (ELISA) in previous studies while we used PEA, an ELISA based array that increases the sensitivity coupling the detection to qPCR.

In this report we found evidence of a possible ongoing inflammation in the CNS of CH patients with elevated levels of several cytokines in the CSF of CH patients as compared to controls. To our knowledge, this is the first reported screening for cytokines in CSF from CH patients. There was very little difference between patients in remission phase and active bout for all the differentially expressed cytokines, which indicates that the neuroinflammatory state in CH patients is not exclusive to the active bout. The cytokines showing the largest increase in CSF from patients were HGF, MMP1, TNSF10 and 12 and several chemokines, all of which were found to have strong interactions in a network analysis. CCL8, CCL11, and CCL13 belong to the CC chemokine family and are primarily involved in attracting leukocytes to sites of inflammation as part of the innate immune system [15]. CCL8, along with other proinflammatory cytokines, have previously been found to be elevated in calvarial periosteum tissue of chronic migraine patients [16]. Experiments on rats have further shown that an inflammatory stimulation of the trigeminal ganglion afferent nerves innervating the calvarial periosteum results in periorbital hypersensitivity, a mechanism potentially relevant for CH, although we found increased CCL8 in CSF and not in serum [17]. Another chemokine, CCL2 has also been found to be elevated in CSF from patients with other primary headache disorders [18]. CXCL10 and CXCL11 are cytokines of the CXC chemokine family, CXC chemokines have chemoattractant properties on leukocytes like the CC chemokines described above, but mainly attract T lymphocytes [19]. Both CXCL10 and 11 bind to the CXCR3 receptor leading to activation of the phospholipase C-dependent pathway, which drives actin rearrangement and increase of intracellular calcium [20, 21]. CXCL10 further plays a role in activation of microglia and migration to sites of lesions or inflammation in the CNS [22, 23]. Considering the broad range of functions and substrates of these proteins, their potential role in CH remains to be clarified. Nociception, intracellular calcium signalling, and microglia activation are all potentially relevant for CH pathophysiology; the first line preventative CH treatment, being a calcium channel blocker, and the top GWAS loci identified for CH, *MERTK*, being primarily expressed in microglia and other glial cells [13, 24].

It is noteworthy that two of the cytokines that were higher in CSF from CH patients, CCL11 and CXCL11, inversely were lower in serum from CH patients compared to controls. A negative correlation between CXCL11 and galectin-3 has previously been found in serum from individuals with ulcerative colitis [25]. Similarly, we found increased concentrations of galectin-3 in serum from CH patients [26]. We also observed a trend for lower levels of CCL13 and CCL7 in serum in the post-hoc analysis, suggesting an overall downregulation of chemoattractant cytokines in serum from CH patients. The opposite direction of regulation of chemokines between CSF and serum is interesting and does not correlate with what is reported for example in multiple sclerosis [27].

Though most elevated cytokines in the CSF of CH patients had a proinflammatory profile, some, such as HGF, are considered as having anti-inflammatory effects [28]. HGF drives cell survival in various cell types and has been known to dampen proinflammatory cytokine release from macrophages [29]. A mendelian randomization study concluded HGF to be a potential causative factor for migraine onset [30]. The HGF-Met pathway has been known to drive differentiation of peptidergic neurons which are especially important in calcitonin gene-related peptide (CGRP) signalling [31]. The importance of CGRP in both migraine and CH pathophysiology suggests a similar mechanism may occur in CH. TNFSF10/tumour necrosis factor-related apoptosis-inducing ligand (TRAIL) and TNFSF12/TNF-related weak inducer of apoptosis (TWEAK), two proapoptotic cytokines in the TNF ligand superfamily were also elevated in CSF in patients. Higher levels of the TNFSF10 receptor, TNFRSF10C, has been found in CH patients during an attack, supporting a role for TNF signalling in CH [6]. The link between TNFSF12 and CH is vague, experimental data supports a potential role for the TNFSF12 receptor, fibroblast growth factor-inducible-14 (Fn14), in neuropathic pain in rodents [32]. MMP1 is part of the matrix metalloproteinase family and are considered immune modulators. Their main function is to break down extracellular matrix, but they also play a role in cytokine release and generation of chemokine gradients [33]. Elevated levels of another member of the MMP family, MMP9, has previously been shown in migraine patients [34]. HGF and MMP1 among other cytokines were also found at higher levels in CH patients in active bout than controls in the posthoc analysis of serum samples. Several of the cytokines identified in the sub-threshold serum analysis are part of the IL-6 superfamily; IL-6, OSM and CSF3. VEGFA was slightly elevated in serum of CH patients and is an interesting candidate as it has repeatedly been shown to have a pro-nociceptive effect [35, 36]. Higher levels of VEGFA have also been found in migraine patients together with elevated CGRP and nitric oxide (NO) [37].

The differences observed between CSF and serum could point towards a greater importance of inflammation in the CNS in the pathophysiology of CH while the peripheral mechanisms have a weaker connection to the immune response. In particular, factors involved in the recruitment of immune cells were upregulated in CSF and we hypothesize that there is a chemoattractant gradient present in these patients, recruiting immune cells and concentrating the inflammation to the CNS. In concordance with our data, previous studies have shown that there is no systemic inflammation in CH patients [5, 38]. However, current data does not provide information on potential local inflammatory reactions that may occur in the peripheral nervous system during CH attacks such as in the trigeminal ganglion.

Initially our hypothesis stated that inflammatory reactions would be specifically upregulated during active bout and may even constitute a hallmark of the phenotypic switch occurring in CH patients when they transition between these two phases. Our results now show a completely different view, demonstrating very similar levels of cytokines between active bout and remission phase. CH patients are typically considered healthy in between bouts, but these data suggest that CH may be considered a chronic disorder, manifesting physiological changes also when patients are in remission. Although published data on CH biomarkers are often conflicting, there are other studies not finding differences in biomarkers between active bout and remission [39, 40], which is in line with our data. It would be of interest to analyse if inflammatory markers normalize with time in elderly patients experiencing long-time/complete remission. The only cytokine specifically related to attacks, IL-13, displayed a trend for increased levels in serum during an attack. IL-13 is typically considered an anti-inflammatory cytokine and is commonly involved in asthma and allergies [41]. It is interesting to note that cultured primary microglia driven to an M1 phenotype have been found to increase their *MERTK* and galectin-3 gene expression in response to IL-13 stimulation, knowing that patients with CH have elevated *MERTK* and galectin-3 in peripheral blood [26, 42]. Due to the difficulties of extracting blood samples during an attack, we had a limited sample size (3) to analyse. Small sample size may lead to both overestimation and underestimation of the effect. In this specific analysis, the small sample size may also be a reason why IL-13 was the sole inflammatory mediator to show a difference in expression levels during an attack. Visual inspection of the data revealed that serum cytokine levels during attacks were highly similar to those of controls, even when there was a difference in patients in active bout or during remission. This normalisation of cytokine levels during attacks has been described for IL-2 in an earlier study [9], and raises questions regarding the underlying mechanisms of the attacks, the cellular origin of the cytokines, and the activation and migration of immune cells in the different phases of the disease. One possibility may be that the CH attack somehow disturbs the chemokine gradient observed between serum and CSF. Unfortunately, we did not have access to CSF from patients during an attack to verify this hypothesis.

OLINK screening of cytokines gives the benefit of performing an unbiased search for immunological markers that may relate to the pathophysiology of CH. Another strength of the study is the large number of markers investigated, maximizing the chances to reveal differentially expressed markers as compared to previous studies on CH investigating specific cytokines. Furthermore, we had the possibility to study cytokines both peripherally and in CSF, so our study provides a more complete view of the immune pathways involved than was previously available. This study also has limitations, including small sample size, which implies that factors that can influence the results (such as age, sex and comorbidities) may be overlooked, and may result in overestimation of the effect. The small sample size may also lead to false negative results because of lack of power, which we attempt to compensate for in our post-hoc analysis exploring sub-significant associations. Also, immune cells have a variety of functions all of which are not completely covered by this OLINK array. In addition, the array does not include any indications of the underlying mechanisms or reveal which cells are responsible for the release of the detected cytokines. Future studies should comprise a broader panel of cytokines and preferably include a larger cohort to increase the power of detecting differences in cytokine levels.

## Conclusion

This study provides the first comprehensive overview of differences in inflammatory markers in CSF and serum of CH patients. Our data clearly show an increase in several inflammatory markers in CSF from CH patients which is suggestive of an inflammation in the CNS. Moreover, differences in chemokines between serum and CSF are indicative of a chemotactic gradient concentrating the inflammation to the nervous system. The inflammation is ongoing regardless of disease status, (active bout or remission).

### Supplementary Information

Supplementary Material 1.

## Data Availability

The datasets generated and/or analysed during the current study are available from the corresponding author on reasonable request.

## Abbreviations

CCL: C–C motif chemokine ligand
CD328: Sialic acid binding Ig like lectin 7
CGRP: Calcitonin gene-related peptide
CH: Cluster headache
CNS: Central nervous system
CSF: Cerebrospinal fluid
CSF3: Colony stimulating factor 3
CXCL: C-X-C motif chemokine ligand
ELISA: Enzyme-linked immunosorbent assay
Fn14: Fibroblast growth factor-inducible-14
GWAS: Genome wide association study
HGF: Hepatocyte growth factor
HLA-DQA1: Major histocompatibility complex, class II, DQ alpha 1
HLA-DQB1: Major histocompatibility complex, class II, DQ beta 1
ICHD: International classification of headache disorders
IFN-γ: Interferon gamma
IL: Interleukin
LOD: Limit of detection
MERTK: MER proto-oncogene, tyrosine kinase
MMP: Matrix metallopeptidase
NF-κB: Nuclear factor- κB
NLRP3: NLR family pyrin domain containing 3
NO: Nitric oxide
NPX: Normalized protein expression
OSM: Oncostatin M
PEA: Proximity extension assay
qPCR: Quantitative real-time PCR
S100: S100 calcium binding protein
TGF-β: Transforming growth factor beta
TNF-α: Tumour necrosis factor alpha
TNFSF: TNF superfamily member
TRAIL: Tumour necrosis factor-related apoptosis-inducing ligand
TSLP: Thymic stromal lymphopoietin
TTH: Tension type headache
TWEAK: TNF-related weak inducer of apoptosis
VEGFA: Vascular endothelial growth factor A
