# Supplementary material for: Elevated cytokine levels in the central nervous system of cluster headache patients in bout and in remission
Source: J Headache Pain. 2024 Jul 23;25(1):121. doi: 10.1186/s10194-024-01829-9 (PMC11267889; doi:10.1186/s10194-024-01829-9)
Supplement: Supplementary file 1 — Supplementary Material 1. [file 10194_2024_1829_MOESM1_ESM.pdf]

Elevated cytokine levels in the central nervous system of cluster headache patients in bout and in remission – SUPPLEMENTARY DATA

Table of contents

Supplementary Table 1:
Cytokine levels in CSF from controls and cluster headache patients 2

Supplementary Table 2:
Cytokine levels in Serum from controls and cluster headache patients 3

Supplementary figure 1:
Assessment of the presence of outliers and normal distribution of data. 4

Supplementary Table 1:
Cytokine levels in CSF from controls and cluster headache patients

	Controls		CH Remission		CH Active Bout				
Cytokine	Mean NPX	Min-Max	Mean NPX	Min-Max	Mean NPX	Min-Max	F Value (df=2)	P-Value	Adjusted P-Value
CCL11	3.02	(1.77-4.42)	3.57	(2.81-4.73)	3.74	(2.58-5.14)	8.71	**<0.001**	**0.003**
CCL13	5.11	(3.92-6.68)	5.87	(4.90-7.18)	5.92	(4.06-7.08)	9.89	**<0.001**	**0.001**
CCL19	11.04	(8.84-13.71)	11.05	(9.11-12.60)	11.12	(8.16-12.63)	0.03	0.973	0.973
CCL2	14.00	(12.61-15.23)	14.39	(13.62-15.16)	14.46	(13.48-15.53)	3.92	**0.025**	0.057
CCL3	6.86	(5.31-8.12)	7.03	(6.09-7.81)	6.85	(6.21-8.09)	0.61	0.545	0.713
CCL4	6.72	(5.71-7.51)	7.09	(5.83-8.08)	6.93	(5.71-7.96)	1.92	0.156	0.251
CCL8	6.50	(4.66-8.53)	7.47	(5.48-8.69)	7.40	(5.56-8.74)	10.42	**<0.001**	**0.001**
CSF1	10.54	(9.13-11.32)	10.87	(10.17-11.61)	10.79	(9.84-11.47)	2.62	0.081	0.157
CSF2	0.99	(0.39-3.10)	1.26	(0.41-1.98)	1.05	(0.30-1.95)	1.43	0.248	0.371
CXCL10	10.05	(8.56-11.68)	10.83	(9.22-12.48)	10.82	(9.10-13.12)	4.96	**0.010**	**0.037**
CXCL11	4.01	(2.60-5.70)	4.77	(2.70-5.75)	4.71	(3.01-7.66)	4.65	**0.013**	**0.043**
CXCL8	11.09	(10.05-12.32)	11.56	(10.17-12.59)	11.55	(10.60-12.39)	4.28	**0.018**	0.053
CXCL9	4.02	(2.19-6.57)	4.50	(3.04-6.34)	4.41	(3.32-6.29)	1.40	0.256	0.371
EGF	0.66	(-0.66-2.88)	1.01	(-0.16-5.97)	0.58	(-0.25-2.22)	1.14	0.326	0.450
FLT3LG	9.43	(8.23-10.90)	9.62	(8.58-10.54)	9.50	(8.61-10.45)	0.57	0.570	0.713
HGF	10.52	(9.33-11.70)	11.64	(10.83-12.29)	11.52	(10.77-12.66)	21.17	**<0.001**	**<0.001**
IL17C	1.43	(0.08-3.97)	1.55	(0.72-2.05)	1.51	(0.45-3.10)	0.19	0.824	0.919
IL18	3.44	(1.37-5.11)	4.05	(3.10-6.29)	3.98	(3.20-4.92)	3.90	**0.026**	0.057
IL6	7.65	(6.84-9.11)	8.16	(6.32-9.80)	8.05	(6.89-9.34)	2.28	0.111	0.190
IL7	1.43	(0.63-2.02)	1.48	(0.44-2.64)	1.58	(0.71-2.82)	0.53	0.590	0.713
LTA	2.36	(0.99-4.76)	2.58	(1.74-3.77)	2.49	(1.41-3.45)	0.47	0.627	0.727
MMP1	5.56	(4.00-7.58)	7.34	(5.88-9.37)	7.18	(5.32-9.48)	19.27	**<0.001**	**<0.001**
MMP12	3.58	(1.19-8.11)	3.43	(1.34-7.28)	3.69	(1.27-7.39)	0.12	0.889	0.954
OLR1	10.59	(8.72-11.72)	11.07	(10.09-11.89)	10.87	(9.92-11.61)	2.98	0.059	0.122
OSM	1.82	(0.60-3.52)	1.81	(0.57-3.18)	1.75	(0.70-2.48)	0.08	0.926	0.959
TGFA	6.20	(4.49-7.39)	6.61	(5.87-7.45)	6.48	(5.68-7.44)	2.32	0.108	0.190
TNFSF10	2.69	(1.00-3.53)	3.24	(2.46-4.46)	3.21	(2.19-4.06)	5.95	**0.004**	**0.018**
TNFSF12	10.86	(9.57-12.15)	11.61	(10.40-12.54)	11.48	(10.39-12.83)	7.51	**0.001**	**0.006**
VEGFA	11.78	(10.36-12.71)	12.30	(11.44-13.29)	12.17	(11.32-12.92)	4.00	**0.024**	0.057

Supplementary Table 2:
Cytokine levels in Serum from controls and cluster headache patients

	Control		CH Remission		CH Active Bout		CH Attack				
Cytokine	Mean	Min-Max	Mean	Min-Max	Mean	Min-Max	Mean	Min-Max	F Value (df=3)	P-Value	Adj. P-Value
CCL11	11.33	(9.14-12.18)	10.35	(8.60-11.74)	10.17	(7.54-11.78)	11.47	(11.39-11.53)	6.05	**0.002**	**0.028**
CCL13	15.30	(13.01-16.37)	14.41	(12.72-16.57)	14.03	(12.10-13.78)	15.42	(15.02-16.08)	4.59	**0.007**	0.053
CCL19	12.48	(11.35-13.45)	12.83	(11.40-14.48)	12.05	(10.71-19.78)	12.87	(12.55-13.05)	3.26	**0.030**	0.105
CCL2	13.50	(12.56-14.38)	13.50	(12.66-14.66)	13.18	(12.22-2.78)	13.22	(13.09-13.33)	1.03	0.387	0.572
CCL3	8.63	(7.90-9.36)	8.71	(7.35-9.91)	8.47	(7.15-3.78)	8.51	(8.26-8.95)	0.42	0.736	0.801
CCL4	9.43	(8.48-10.44)	9.77	(8.77-11.30)	9.33	(7.97-4.78)	9.23	(8.77-9.79)	1.25	0.302	0.489
CCL7	5.80	(3.26-6.80)	5.57	(4.27-6.93)	4.91	(2.83-7.78)	5.75	(4.34-6.68)	2.65	0.060	0.172
CCL8	11.12	(9.99-11.78)	11.32	(10.70-12.07)	10.84	(7.36-8.78)	11.28	(10.94-11.87)	0.85	0.474	0.650
CSF1	11.35	(11.12-11.70)	11.32	(10.72-11.65)	11.21	(10.56-1.78)	11.13	(10.97-11.31)	1.25	0.304	0.489
CSF2	3.63	(2.79-4.96)	3.82	(2.89-4.53)	3.32	(1.90-2.78)	3.23	(2.95-3.52)	2.39	0.081	0.200
CSF3	4.59	(3.80-5.23)	5.66	(4.49-9.11)	4.84	(3.53-3.78)	4.68	(4.46-5.07)	4.55	**0.007**	0.053
CXCL10	11.29	(9.83-13.16)	11.34	(10.57-12.62)	10.75	(9.74-10.78)	10.79	(9.05-12.17)	1.61	0.201	0.391
CXCL11	12.32	(7.68-15.53)	10.36	(7.93-13.34)	9.90	(7.43-11.78)	12.54	(10.22-13.97)	6.60	**0.001**	**0.028**
CXCL8	8.58	(7.87-9.89)	8.94	(8.31-10.12)	8.91	(7.30-8.78)	8.59	(8.16-9.45)	0.86	0.469	0.650
CXCL9	9.13	(7.60-12.32)	9.06	(7.99-10.27)	8.37	(7.27-9.78)	8.89	(7.72-9.64)	2.21	0.100	0.226
EGF	9.18	(7.73-12.10)	9.00	(6.87-11.55)	9.74	(6.44-.78)	9.13	(7.93-10.17)	0.43	0.733	0.801
FLT3LG	10.37	(9.64-11.58)	10.52	(9.92-11.22)	10.25	(8.32-3LG.78)	10.17	(9.73-10.63)	0.59	0.622	0.767
HGF	10.10	(9.18-11.31)	11.00	(9.48-12.28)	10.76	(9.57-.78)	9.89	(9.35-10.35)	5.07	**0.004**	0.051
IFNG	5.31	(3.38-7.74)	4.94	(3.93-6.97)	4.85	(3.43-G.78)	5.15	(3.88-6.27)	0.60	0.618	0.767
IL10	6.63	(5.72-7.95)	6.40	(5.24-7.33)	6.34	(4.57-10.78)	6.45	(6.25-6.62)	0.74	0.532	0.704
IL13	2.08	(1.06-6.88)	2.08	(1.10-3.70)	1.78	(1.07-13.78)	4.32	(1.73-7.70)	3.60	**0.020**	0.084
IL17C	5.54	(3.32-7.04)	4.95	(4.28-6.42)	4.77	(3.67-17C.78)	5.34	(4.99-5.60)	3.02	**0.040**	0.122
IL18	10.64	(9.61-12.43)	10.78	(9.61-11.59)	10.60	(9.12-18.78)	10.34	(10.02-10.59)	0.36	0.783	0.828
IL2	1.48	(0.93-2.52)	1.26	(1.03-1.73)	1.32	(0.90-2.78)	1.24	(1.07-1.57)	1.03	0.386	0.572
IL27	6.18	(1.90-9.25)	6.58	(1.89-8.82)	6.78	(1.78-27.78)	5.72	(3.47-8.19)	0.29	0.830	0.853
IL6	7.08	(6.09-8.39)	8.33	(6.13-11.26)	7.48	(5.98-6.78)	7.95	(6.58-9.63)	3.77	**0.017**	0.079
IL7	2.95	(1.66-5.02)	3.78	(2.13-5.90)	3.68	(1.53-7.78)	2.49	(2.13-3.01)	2.18	0.104	0.226
LTA	6.37	(5.84-8.21)	6.40	(5.29-7.40)	6.28	(5.65-.78)	6.21	(5.77-6.50)	0.22	0.879	0.879
MMP1	12.22	(11.03-13.92)	13.35	(11.21-15.21)	13.18	(10.76-1.78)	11.97	(10.71-13.54)	4.01	**0.013**	0.076
MMP12	8.72	(7.21-11.10)	9.12	(7.82-10.44)	8.57	(7.04-12.78)	9.44	(9.32-9.61)	1.49	0.230	0.404
OLR1	8.89	(7.47-11.56)	9.53	(7.61-11.94)	9.42	(6.96-1.78)	7.89	(7.80-7.97)	1.51	0.225	0.404
OSM	4.94	(3.13-8.00)	6.54	(3.97-8.63)	6.32	(2.81-.78)	4.62	(4.05-5.62)	3.92	**0.014**	0.076
TGFA	2.80	(2.29-3.38)	3.24	(1.96-5.25)	2.87	(1.93-A.78)	2.63	(2.43-2.85)	2.02	0.125	0.257
TNF	4.64	(3.75-5.26)	4.30	(2.72-5.26)	4.11	(2.70-.78)	4.56	(4.47-4.71)	2.46	0.075	0.199
TNFSF10	9.38	(8.79-9.76)	9.52	(9.00-10.07)	9.36	(8.36-10.78)	9.37	(9.24-9.63)	0.46	0.709	0.801
TNFSF12	10.23	(9.65-10.78)	10.14	(9.29-11.19)	10.04	(8.81-12.78)	9.94	(9.67-10.34)	0.50	0.681	0.801
VEGFA	12.11	(11.38-12.90)	12.77	(11.53-14.78)	12.39	(10.94-FA.78)	12.04	(11.70-12.34)	3.22	0.031	0.105

Supplementary figure 1:
Assessment of the presence of outliers and normal distribution of data.

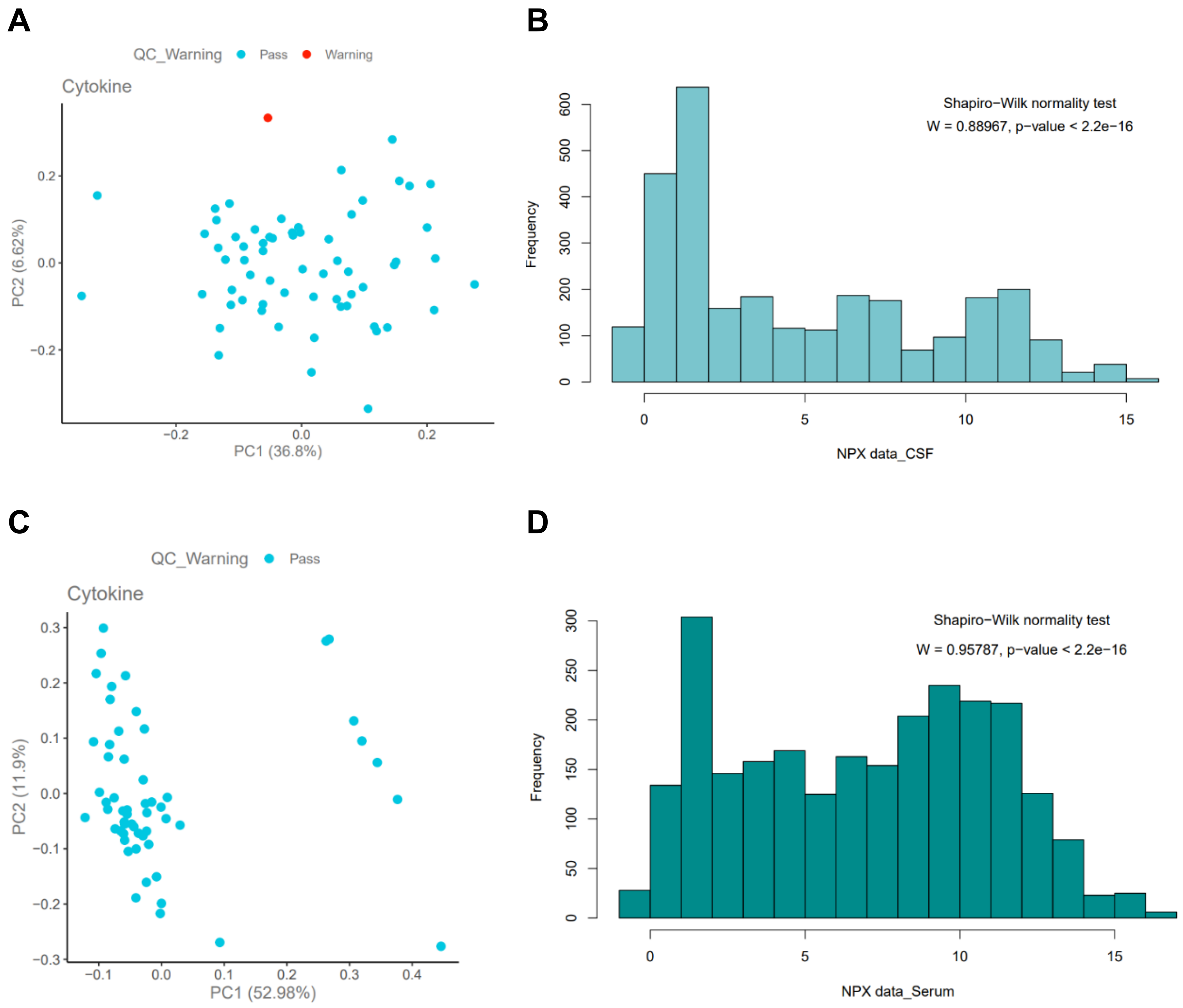

Left panel: PCA analysis of NPX data from serum samples (A) and CSF samples (C) from cluster headache patients and controls revealed seven potential outliers among CSF samples. Right panel: Distribution of NPX data from serum samples (B) and CSF samples (D) was non normal.
